# Editorial: Developmental Abnormalities of Serotonin Homeostasis in Behavioral and Metabolic Disorders: From Epigenetic Mechanisms to Protein Function

**DOI:** 10.3389/fnins.2021.659356

**Published:** 2021-05-13

**Authors:** Dubravka Hranilovic, Jasminka Stefulj, Peter Zill

**Affiliations:** ^1^Division of Animal Physiology, Department of Biology, Faculty of Science, University of Zagreb, Zagreb, Croatia; ^2^Laboratory of Neurochemistry and Molecular Neurobiology, Department of Molecular Biology, Rudjer Boskovic Institute, Zagreb, Croatia; ^3^Department of Psychiatry and Psychotherapy, University Hospital, LMU Munich, Munich, Germany

**Keywords:** adverse environment, gender-specific regulation, 5-HT transporter, obesity, animal model

Serotonin (5-hydroxytryptamine, 5-HT) is a phylogenetically ancient signaling molecule with pleiotropic effects in vertebrate organisms (Nardi et al., 2017). In the central nervous system, it regulates developmental processes (Kepser and Homberg, 2015), and modulates integrative functions and states, such as mood, anxiety, stress, aggression, sexual behavior, and cognition (Olivier, 2014). Outside the nervous system, it exerts hormonal and paracrine/autocrine actions by activating 5-HT receptors in various somatic tissues (Amireault et al., 2013). Both, central and peripheral serotonin act on multiple metabolic organs in order to maintain systemic energy homeostasis (Donovan and Tecott, 2013; Yabut et al., 2019). Although serotonin has been widely studied as a suspect in behavioral and metabolic disorders, the relationship between 5-HT disturbances in peripheral and central compartments has not been completely resolved, the causal role of the observed 5-HT alterations in the etiology of various disorders has not been fully established, and the molecular substrates of improper expression and function of proteins regulating 5-HT signaling have not been entirely identified. These issues are addressed in the current Research Topic by five papers employing various research methods on different vertebrate organisms, with a common goal of untangling the link between developmental abnormalities of serotonin homeostasis and behavioral and/or metabolic alterations in adult life.

Keeping in mind that 5-HT has a prominent role in prenatal programing of the hypothalamo–pituitary–adrenal axis, Vera-Chang et al. explored the hypothesis that exposure to the 5-HT reuptake blocker fluoxetine (FLX), a widely used antidepressant, during the early or late period of sexual development impairs the stress axis of adult zebrafish (*Danio rerio*), consequently disrupting their behavioral responses in a sex- and time-dependent manner. Significantly lower stress-induced whole-body cortisol levels were observed in FLX-treated vs. control animals, and in females vs. males. In addition, late FLX-treated females showed increased, while early FLX-treated males showed decreased exploratory behavior compared to controls of the same sex. The results of this study point to the importance of sex- and timing-based approaches to FLX treatment, warn of the possible behavioral effects of FLX on adults exposed during fetal development, and show the potential impact of FLX on aquatic species in environments receiving sewage effluents.

Building upon evidence of the involvement of 5-HT disfunction in the etiology of autism spectrum disorders (ASD), Chakraborti et al. investigated the relationship between blood 5-HT parameters and severity of behavioral symptoms, as well as gender effect on both measures. Lower platelet levels of 5-HT and its metabolite 5-hydroxyindole acetic acid (5-HIAA) in control males compared to females, a male-specific increase in 5-HT measures correlating with increased intellectual disability in ASD probands, and the presence of milder ASD symptoms in males than in females suggest higher vulnerability and a lower threshold for behavioral expression of ASD in boys. The study highlights a gender-specific regulation of platelet 5-HT homeostasis and its differential contribution to behavioral expression of ASD in males and females, partially explaining the higher male to female incidence ratio in ASD.

The paper by Veniaminova et al. places serotonin at the crossroad of mental and metabolic conditions by investigating the mechanism through which the reduced function of the serotonin transporter (SERT or 5-HTT) contributes to the development of anxiety, depression, and type-2 diabetes, especially in elderly women. The study is focused on the metabolic, molecular, and behavioral consequences of a 3-week exposure of aged female mice with complete (*Sert*^−/−^) or partial (*Sert*^+/−^) SERT gene inactivation to a fat- and sugar-rich diet (“Western Diet,” WD). All genotypes challenged with WD displayed weight gain and depressive-like behavior. Interestingly, decreased glucose tolerance, overexpression of a neuroinflammation marker, and disrupted hippocampus-dependent performance, found in the WD-fed wild-type mice, were exacerbated in *Sert*^−/^^−^ mice, but absent in *Sert*^+/−^ mice. The results reveal a complex interplay between genetic makeup (SERT abundancy/function) and environmental factors (type of diet) in regulating metabolism and behavior during aging. In addition, the observed resilience of *Sert*^+/^^−^ mice to certain environmental challenges supports the concept of heterosis as an evolutionary adaptive mechanism.

The study of de Lima et al. starts with the premise that synergy between the altered 5-HT signaling and adverse environmental factors in early childhood increases a risk for behavioral disorders. The authors used a unique approach—they identified genes co-expressed with 5-HTT in the amygdala (an emotion-processing brain region) during brain development and named them a “5-HTT gene network.” They calculated a polygenic risk score reflecting the function of the 5-HTT gene network, and studied its interaction with postnatal adversity in order to predict genome methylation status (a link between environmental factors and gene expression), cortical density (a link between expression and function), and attention deficit hyperactivity disorder (ADHD)-related symptoms (behavioral outcome presumably underlaid by 5-HT alterations) in pre-school children. They showed that exposure to postnatal adversity and decreased function of the 5-HTT gene network were associated with negative behavioral outcomes and altered methylation status throughout the genome, and that the 5-HTT gene network affected gray matter density in brain regions linked to attentional processes. By revealing the association of the 5-HTT gene network with vulnerability or resilience to environmental factors, and indicating the mechanism by which adversity exerts its effects on attention/hyperactivity, the results may have implications for both prevention and therapy of ADHD.

A growing body of evidence highlights the important contribution of 5-HT to obesity and represents the starting point of the study by Kesić et al. which explores the mechanism of this relationship. The authors studied physiological phenotypes of body weight homeostasis and hypothalamic expression of body weight–regulating molecules in sublines of rats that are constitutionally hyper- (high-5HT) or hypo- (low-5HT) serotonergic. High-5HT animals consumed more food, displayed a significant increase in body mass, fat amount, and inner organ weight, as well as increased hypothalamic expression of neuropeptide Y receptor and a trend for upregulation of other orexigenic molecules. The study shows that 5-HT is one of the endogenous factors contributing to, or protecting from, becoming obese, presumably by acting both, as a peripheral (by controlling glucose/lipid metabolism) and a central (by regulating feeding) signal.

Taken together, the presented papers identified some of the mechanisms by which developmental disruption of 5-HT homeostasis, through its complex interplay with adverse environmental factors and gonadal hormones, increases vulnerability to behavioral and metabolic disorders. These findings open new avenues of research and set paths for the improvement of early diagnostics and individualized therapy.

## Author Contributions

DH wrote the first draft, JS and PZ provided critical discussion and participated in the draft finalization.

## Conflict of Interest

The authors declare that the research was conducted in the absence of any commercial or financial relationships that could be construed as a potential conflict of interest.
